# Low COST surgery setting for one-operational port laparoscopic hysterectomy surgery with ordinary laparoscopic instruments: preliminary results

**DOI:** 10.1186/1750-1164-7-13

**Published:** 2013-10-02

**Authors:** Leo Francisco Limberger, Luciana Silveira Campos, Nilton Jacinto Rosa da Alves, Daniel Siqueira Pedrini, Andiara Souza de Limberger

**Affiliations:** 1 Hospital Nossa Senhora da Conceição, Porto Alegre, RS, Brazil; 2 Hospital Moinhos de Vento, Porto Alegre, RS, Brazil; 3 Universidade Luterana do Brasil, Canoas, RS, Brazil

**Keywords:** Videolaparoscopy, Hysterectomy, Single port

## Abstract

**Background:**

Hysterectomy dates back to 120BC and is the second most commonly performed gynecological surgery in the world. Cosmetic demands and the necessity of rapid return to work have contributed to the minimally invasive laparoscopic approach for hysterectomy. The majority of reports describe the use of three or four incisions to perform the surgery (two or three for manipulation and one for optics).

**Methods:**

This work describes our experience with using only two ports for 11 patients who underwent video-laparoscopic hysterectomy surgery. One port was used for the optical system, and the second was used for manipulation. Early and late surgery complications, as well as the time to return to work and daily activities, were assessed.

**Results:**

The mean age of the patients was 41.4 years old (range 16 to 52 years) and the mean uterine weight was 133.54 g, ranging from 35 g and 291 g. The operative time ranged from 30 to 60 minutes (average 46.4 minutes) and the hospital stay ranged between 24 and 48 hrs. No intraoperative complications occurred, and no early or late postoperative complications were recorded. Patients reported minimal pain during the first 24–48 hrs in the hospital. Patients returned to their daily activities within seven days after surgery. Clinical care follow-up continued until the 40th postoperative day.

**Conclusion:**

The laparoscopic hysterectomy technique with a single port for manipulation is a feasible procedure when the uterine weight is not greater than 400 mg with little postoperative pain. The patients had an early return-to-work and daily activities and a better cosmetic outcome. These preliminary data led us to make the one-operative port laparoscopic hysterectomy the procedure of choice for patients with a low uterine weight.

## Background

Hysterectomy is the second most common gynecological surgery after cesarian delivery, with comparatively rare serious complications. Currently, hysterectomy is the second most common surgical procedure in America with 650000 to 700000 procedures being performed annually [1]. The mean age of the patients that undergo hysterectomy is 47 years old. At the age of 55 and 60, 25 and 30% of the American women had their uterus removed, respectively [2]. Majority of the hysterectomies are performed for benign indications such as menorrahgia, leiomyomatosis and adenomyosis, which respond to nearly 75% of all abdominal hysterectomies. Uterine prolapsed corresponds to 80 to 90% of vaginal hysterectomies [1].

The introduction of laparoscopy as a new approach for hysterectomy brought up a new understanding of gynecological surgery as a whole. New surgical methods to section and to suture, as well as new instruments and a better anatomy understanding contributed to considerable advances in the field. All these changes made gynecologists reformulate their principles and renew their surgical procedures [3]. Additionally, we live in a time that body image is greatly valued, in a constant search for the “perfect body” and, at the same time, patients demand a fast recovery and return to work and normal daily activities after the surgical procedure [4]. One major step forward in the laparoscopic surgical methodology happened with the introduction of videolaparoscopy, which allowed access to the abdominal cavity with more accuracy, less risks to the patients and reduced complications on cosmesis and indisputable better cosmetic results [5].

In this scenario, laparoscopy became the first choice procedure for most women, in case they have an indication of uterine removal. Global access to information showed to women that they can have their uterus removed in a less traumatic approach, with a speedy recovery, less pain and minimal stay in hospital together with an excellent cosmetic outcome [6].

Traditionally, videolaparoscopy requires three or four laparoscopic ports – an optical port and two or three ports for the manipulations being that the UTERUS is removed through the vagina. The choice of method to be used usually depends on the surgeon’s training. Thus, we consider the vaginal hysterectomy a more difficult procedure than the abdominal approach. Although laparoscopic hysterectomy offers a better inspection, it is more difficult to perform than the vaginal route, it requires more training, a good agreement among the team members and a sophisticated and unique set of instruments [7].

A study published in Texas reported that the advent of laparoscopic hysterectomy increased the rates of vaginal hysterectomy from 27.7 to 53.2% and decreased the rates of abdominal laparoscopies in 29% [8]. The choice of method to be used usually depends on the surgeon’s training. In this report, we describe our experience in using two ports for videolaparoscopic hysterectomy – one for optics and one for manipulation. The main outcome variables analyzed were operative time, length of hospital stay, postoperative patient comfort, return to normal activity as reported by the patients and cosmetic results.

## Methods

The study was approved by the Ethics Committee of the Hospital Nossa Senhora da Conceição, a public tertiary hospital, where the surgeries were performed, between October 2008 and September 2011. We describe eleven patients, who underwent videolaroscopic hysterectomy performed by one surgeon, using a single-operative laparoscopic port of 10 mm for the optics and another of 5 mm for the manipulations. Inclusion criterion for the study was a planned hysterectomy for different gynecologic conditions. Exclusion criteria were uterine weight greater than 400 g, as evaluated by transvaginal pelvic ultrasound and the presence of any clinical or ultrasonographic signs of previous pelvic infections or cardiac conditions that were not compatible with the laparoscopy.

Ten patients were referred from a private clinic and one from a tertiary referral public hospital. The newness of the operative procedure to be used and the potential complications were thoroughly explained to the patients, and they provided written informed consent for the surgery. The indications for hysterectomy were abnormal uterine bleeding, unresponsive to hormone therapy or NSAIDs prescription and leiomyomatosis, adenomyosis, confirmed by transvaginal pelvic ultrasonography and that and could not be managed with conservative therapy and one *in situ* cervical carcinoma.

**Figure 1:**
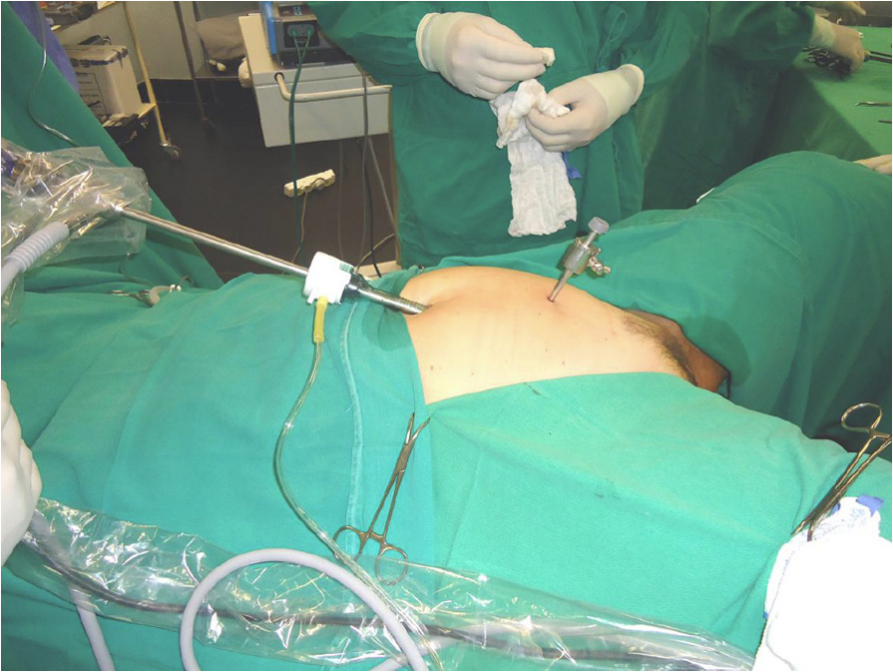
Photograph showing the one-operative port videolaparoscopic hysterectomy.

The operation procedures started with the upper members fixed along the body. Patients received a combination of spinal and IV general anesthesia. Vesical catheterization was then performed with a #18 catheter, and a uterine manipulator with chrome-plated tubing was put in place. Insufflation using a Veress needle and supraumbilical puncture with a 10-mm trocar for a 30° optical introduction was performed. Insufflation started with 1 L/min until the introduction of 2 L of CO_2_ into the cavity, when the maximum insufflation possible was reached with the device. We worked with pressures ranging between 15 and 18 mm Hg, according to patient tolerance. Next, we performed a systematic inspection of the peritoneal cavity followed by the performance of another 5-mm suprapubic puncture. (Figure 1) Sealing and sectioning of the the round ligament and sealing and section of infundibular pelvic ligament. Uterine vessels were sectioned and sealed after the vesicouterine peritoneum was dissected with rhomboid detachment of the bladder, this gauze was introduced via the optical trocar and removed by the vaginal opening. Cephalad impulsion of the uterus and separation of the bladder from the vaginal wall was performed contralaterally. The vagina was circumferentially transected and extracting tweezers were applied vaginally for removal of specimen. Maintenance of the pneumoperitoneum was performed by vaginal tamponing with glove fingers filled with saline. The vaginal cuff was sutured through the vaginal route.

Four out of the eleven surgeries were performed using ultracision blades (Harmonic® Ultrasonic, Ethicon Endo-Surgery), one used auto-sonix blade (Harmonic Autosonix; Codien), one used ENSEAL® TRIO (Ethicon Endo-Surgery), and five used bipolar blade, and scissors. Surgeries were performed exclusively by laparoscopy and the morcellated uterus was removed through the vagina. A 3–0 poliglactin was used to close the vaginal cuff. The adjacent organs were preserved in all procedures.

## Results

The mean age of the patients was 41.4 years old (range 16 to 52 years) and they did not present any comorbidity. The mean uterine volume was 133.54 g, ranging from 35 and 291 g.

Operative time from skin incision to skin closure ranged from 30 to 60 minutes (average 46.4 minutes). Hospital stay ranged between 24 and 48 hrs, being that one woman was discharged 18 hrs postoperative. No intraoperative complications occurred and no early or late postoperative complications were recorded. Patients reported minimal pain during the first 24–48 hrs in hospital. Patients returned to daily activities within seven days after surgery. The clinical care follow-up continued until the 40th postoperative day (Additional file 1).

## Discussion

The results of our study show an excellent postoperative outcome in terms of recovery time, hospital stay, patient comfort and cosmetic results, when the one-operative port approach for laparoscopic hysterectomy was used. Several studies have demonstrated that laparoscopic hysterectomy is less invasive and the postoperative recovery is faster than open surgery, possibly, because the wounds are smaller [9,10].

In our study, patients reported a fast return to normal activities and little or no pain 24-48 hrs after the hysterectomy procedure here described. The reported comfort and reduced pain allowed a fast return to daily activities. These positive outcomes may be explained by the size of the wounds required for the surgical procedure, as elegantly described by Blinman [11] and by Carvalho & Cavazzola [12], who described that conventional large incisions result in more morbidity, than using two small trocars. Furthermore, the results achieved with the present series of patients compare favorably with recent reports using the latest robotic technology, where a significant rate of intra- or post-operative complications were described [13].

One major point to emphasize in the present report is the fact that the one-operative port hysterectomy method here described does not demand any special surgical instruments, neither sophisticated hospital settings allowing it to be performed in any hospital that already does routine videolaparoscopic procedures of medium complexity. Also, the methodology can be successfully applied to the majority of the uterine pathologies requiring no special training in performing the surgery, provided that the surgeon has already overcome the steep learning curve traditionally needed for laparoscopic hysterectomy. We propose this technique as an option to three-port laparoscopic hysterectomy, since laparoscopic hysterectomy presented lower pain scores compared to vaginal hysterectomy in a metaanlysis” (weighted mean difference: - 2.13; 95% CI: - 4.08 a - 0.18; P = 0.03)” [14].

From the point of view of the patient population, this new approach presents a favorable perception of a scarless surgery, reduced pain and stay in hospital, together with good cosmesis.

Here we demonstrated the factibility of one-operative port hysterectomy with ordinary surgical material. We believe that the method described in this report is a feasible and safe procedure, with low costs and without the need of sophisticated skills by the medical staff. It can benefit a large population of patients attending to public hospitals, particularly in low income settings. Further studies are required to compare one-operative port hysterectomy with the usual three or four trocars approach.

## Conclusion

The videolaparoscopic hysterectomy technique with a single port for manipulation is a valid and feasible procedure, when uterine weight is not greater than 400 mg. The methodology showed relevant results in terms of reduced bleeding and portal complications, as well as little postoperative pain. The patients had an earlier return to work and daily activities and an undisputable better cosmetic outcome.

More cases must be performed to consolidate our results. However, these preliminary data led us to make the one-operative port videolaparoscopic hysterectomy the choice procedure to treat patients with low uterine weight.

## Competing interest

The authors declare that they have no competing interest.

## Authors’ contributions

LFL: performed the surgeries as the first surgeon, designed the protocol, followed the patients and drafted the manuscript. LSC: designed the protocol, analysed data and drafted the manuscript. NJRA: designed the protocol, performed the surgeries as the second surgeon. DPS: designed the protocol and followed the patients ASL: designed the protocol, colleted and analysed data. All the authors have read and approved the final manuscript.

## Supplementary Material

Additional file 1 Patients’ characteristics. Click here for file
